# Effect of body mass and clothing on decomposition of pig carcasses

**DOI:** 10.1007/s00414-014-0965-5

**Published:** 2014-02-02

**Authors:** Szymon Matuszewski, Szymon Konwerski, Katarzyna Frątczak, Michał Szafałowicz

**Affiliations:** 1 Laboratory of Criminalistics, Adam Mickiewicz University, Św. Marcin 90, 61-809 Poznań, Poland; 2 Natural History Collections, Adam Mickiewicz University, Umultowska 89, 61-614 Poznań, Poland; 3 Department of Animal Taxonomy and Ecology, Adam Mickiewicz University, Umultowska 89, 61-614 Poznań, Poland

**Keywords:** Forensic entomology, Forensic taphonomy, Carrion decomposition, Processes in decomposition, Total body score, Carcass mass loss, Post-mortem interval

## Abstract

Carcass mass and carcass clothing are factors of potential high forensic importance. In casework, corpses differ in mass and kind or extent of clothing; hence, a question arises whether methods for post-mortem interval estimation should take these differences into account. Unfortunately, effects of carcass mass and clothing on specific processes in decomposition and related entomological phenomena are unclear. In this article, simultaneous effects of these factors are analysed. The experiment followed a complete factorial block design with four levels of carcass mass (small carcasses 5–15 kg, medium carcasses 15.1–30 kg, medium/large carcasses 35–50 kg, large carcasses 55–70 kg) and two levels of carcass clothing (clothed and unclothed). Pig carcasses (*N* = 24) were grouped into three blocks, which were separated in time. Generally, carcass mass revealed significant and frequently large effects in almost all analyses, whereas carcass clothing had only minor influence on some phenomena related to the advanced decay. Carcass mass differently affected particular gross processes in decomposition. Putrefaction was more efficient in larger carcasses, which manifested itself through earlier onset and longer duration of bloating. On the other hand, active decay was less efficient in these carcasses, with relatively low average rate, resulting in slower mass loss and later onset of advanced decay. The average rate of active decay showed a significant, logarithmic increase with an increase in carcass mass, but only in these carcasses on which active decay was driven solely by larval blowflies. If a blowfly-driven active decay was followed by active decay driven by larval *Necrodes littoralis* (Coleoptera: Silphidae), which was regularly found in medium/large and large carcasses, the average rate showed only a slight and insignificant increase with an increase in carcass mass. These results indicate that lower efficiency of active decay in larger carcasses is a consequence of a multi-guild and competition-related pattern of this process. Pattern of mass loss in large and medium/large carcasses was not sigmoidal, but rather exponential. The overall rate of decomposition was strongly, but not linearly, related to carcass mass. In a range of low mass decomposition rate increased with an increase in mass, then at about 30 kg, there was a distinct decrease in rate, and again at about 50 kg, the rate slightly increased. Until about 100 accumulated degree-days larger carcasses gained higher total body scores than smaller carcasses. Afterwards, the pattern was reversed; moreover, differences between classes of carcasses enlarged with the progress of decomposition. In conclusion, current results demonstrate that cadaver mass is a factor of key importance for decomposition, and as such, it should be taken into account by decomposition-related methods for post-mortem interval estimation.

## Introduction

Although much progress was made in the field of methods for post-mortem interval (PMI) estimation, particularly in forensic entomology [1–3] and forensic taphonomy [4–6], our understanding of carrion decomposition and related biological processes is still insufficient which hinders further progress in these fields [7]. Carrion decomposition is a complex mix of processes, influenced by many biotic and abiotic factors [8]. Accordingly, any decomposition-related approach to PMI estimation should take into account effects of these factors. Unfortunately, our understanding of these effects is still very poor, some were not tested at all and the others were tested improperly; even in the case of those which were extensively studied, results of different studies are conflicting or incomplete. Therefore, there is still a need for basic research in the field of carcass decomposition.

Carcass mass and carcass clothing are factors of probable high forensic importance. In casework, corpses are of different mass and different kind or extent of clothing. Accordingly, if these factors affect decomposition, methods of PMI estimation should take it into account.

Based on experience and case studies, Mann et al. [8] created a five-point scale for the effect of various factors on the decay rate of a human body. Within this approach body size and weight were scored 3, whereas corpse clothing was scored 2. Authors suggest that body mass is a factor of lesser importance than it would logically seem to be and support this statement with two examples of 110-kg bodies which decayed more rapidly than bodies weighing about 65 kg [8]. In a review article, Marchenko indicates that the influence of cadaver mass on its decay period depends on meteorological factors, whereas clothes on a cadaver make the decay period somewhat longer [9].

First experiments on the importance of the carcass size (mass) and type were undertaken within the ecology of carrion insect communities [10, 11]. However, these studies were designed to analyse just the composition and structure of carrion fly communities, and not the progress of decomposition. Moreover, carcasses being compared were all much below 1 kg, so definitely beyond the scope of forensic relevance.

In the first forensic study, Hewadikaram and Goff [12] compared decomposition of 8.4 and 15.1-kg pig carcasses, demonstrating a more rapid rate of decomposition in a larger carcass. This view was challenged by the results of Komar and Beattie [13] from an experiment with small (19–26 kg), medium (36–80 kg) and large (156–162 kg) clothed pig carcasses decomposing in shaded and sunlit habitats. It was found that smaller carcasses decayed significantly faster than medium and large carcasses in both types of habitat, suggesting that the small-sized carcasses are not an appropriate model for human decay rates [13]. More recently, Brand [14] compared the decomposition rate of 10, 15 and 20-kg pig carcasses revealing no significant difference in total body score between these classes of carcasses. However, a meta-analysis of Simmons et al. [15], which included a broader range of carcass mass, demonstrated that this factor is of key importance for the rate of decomposition, but only in the case of these corpses which were colonized by insects. Results of Simmons et al. [15] confirmed previous view that small carcasses decompose faster than large carcasses. This view was also confirmed by an experiment of Spicka et al. [16] with ~1, ~20, ~40 and ~50-kg pig carcasses. These authors have also found that carcass decomposition follows a sigmoidal curve irrespective of carcass mass and that mass loss is faster in neonatal and ~20-kg carcasses as compared to larger carcasses [16]. The last experiment, made in the temperate region of South Africa by Sutherland et al. [17], compared decomposition of small (<35 kg) and large pig carcasses (60–90 kg). It revealed that small carcasses decompose about 2.8 times faster than large ones. Large corpses showed a plateau phase (with minimal decomposition) in the course of advanced decay, small carcasses weighing more than 20 kg revealed a shorter plateau phase, whereas small corpses weighing less than 20 kg revealed no plateau phase and a swift decomposition throughout the study [17].

Clothing attracted less attention of researchers. Although its importance is recognized in the field [18], little studies were devoted to analyse its effects on decomposition. While studying the influence of clothing and wrapping on decomposition and arthropod succession, Kelly et al. [19] demonstrated that carcass wrapping is of clear importance, as it results in larger, more visible and more freely moving larval masses, longer duration of advanced decay and more biomass remaining after termination of active decay. However, clothing (considered in isolation) was found to be of no importance, since control and clothed carcasses decomposed with the same pattern and rate as control and unclothed carcasses [19]. In a 2-year study of pig carrion decomposition and insect succession in Western Australia, Voss et al. [20] challenged this result, revealing that the wet decay stage is markedly prolonged in clothed as compared to unclothed pig carcasses. Moreover, dipteran larval masses were more widely distributed across the surface of clothed carcasses and were present for a longer period of time on these carcasses [20].

This review leads to a conclusion that carcass mass influences decomposition rate and that small carcasses decompose faster than large ones. However, in a low mass range (carcasses below 20 kg), strength of the relationship and even its kind are unclear. Existing results are conflicting, with some indicating a negative relationship between carcass mass and decay rate [16, 17] and others suggesting no relationship [14] or even a positive relationship [12]. Accordingly, a question arises whether decomposition rate responds uniformly in a full range of masses or differently in a range of small and large masses. Moreover, it is not clear which processes during decomposition are affected by carcass mass. Results of Simmons et al. [15] suggest that carcass mass affects only insect-driven processes and specifically active decay as defined by Matuszewski et al. [21]. The problem, however, needs to be directly investigated. As for the effect of clothing, existing results are obviously conflicting. Accordingly, the importance of clothing is still unclear and further experiments are needed.

In this article, we analyse simultaneously the effects of carcass mass and clothing on particular decompositional processes and related phenomena, based on the results of a factorial block experiment with a broad range of carcass masses (5–70 kg) and full clothing of carcasses included.

## Materials and methods

### Experimental design

**Fig. 1:**
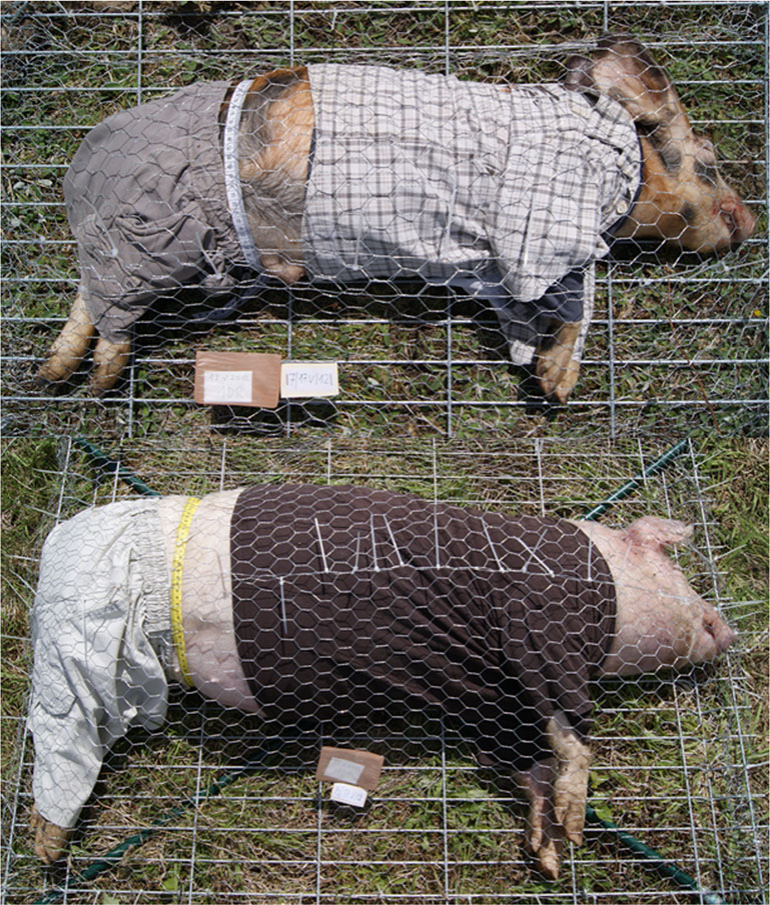
The kind of clothing used during the experiment

The experiment was made according to the complete factorial block design. Factors of interest were carcass mass and carcass clothing. The study included four levels for carcass mass (small carcasses 5–15 kg, medium carcasses 15.1–30 kg, medium/large carcasses 35–50 kg and large carcasses 55–70 kg) and two levels for carcass clothing (clothed and unclothed). Clothed carcasses were dressed in trousers (with shortened legs), t-shirt and a shirt or a blouse (Fig. 1). All clothes were made of cotton and were of similar colours. Carcasses were grouped into three blocks which were separated in time (the day of carcass exposition: 17 May, 16 July and 27 August 2012). Within each block, all levels for both factors were studied. In total, 24 carcasses were used, eight in each block.

The study was made in xerothermic grasslands of the Biedrusko military range (Western Poland, Europe). This is a rural area covered with grasses with no trees or bushes.

In each block, eight similar sites were chosen along a roughly straight line. Adjacent sites were far away from each other by at least 50 m. Carcasses were randomly assigned to the sites.

### Carcasses

In total, 24 domestic pig carcasses were bought from a local pig farm. Pigs were killed at about 6 a.m. (a blow to the base of the skull) and, after 1 to 3 h, were exposed in the field. Carcasses were laid down on a metal grate and were protected with welded wire mesh.

### Monitoring of decomposition and sampling of insects

Carcasses were inspected once a day until about the 20th day post-mortem. Afterwards, examinations were less frequent (every second, third, fourth, fifth and seventh day). Inspections were made by two examiners and lasted usually about 30 min per carcass (between 10 a.m. and 2 p.m.).

The progress of decomposition was documented by using a camera and written reports. Gross processes in decomposition (bloating, active and advanced decay) were monitored, and their onsets and durations (as defined by Matuszewski et al. [21]) were recorded. Bloating was quantified by measuring girth of the abdomen near the back legs of a carcass. For this purpose, carcasses were permanently girthed with plastic measuring tapes. Carcasses were weighed by using the hanging scale KERN HCB 99 K50 (KERN & Sohn, Balingen, Germany). A carcass (laying on a metal grate) was suspended to a scale, which was hanged on the metal or wooden stake. In order to weigh the carcass, examiners picked up a stake. Moreover, decomposition was scored according to the total body score (TBS) system of Megyesi et al. [22].

Insects were sampled using standardized protocols. Two pitfall traps per carcass filled with 50 % glycol solution were used. Manual collections were taken from the carcass surface and from the soil under and near the carcass. Flying insects were sampled with a large aerial sweep net.

### Temperature measurements

Ground level temperature was measured at every carcass. For this purpose, HOBO U23 Pro v2 2× External Temperature Data Loggers (Onset Computer Corporation, MA, USA) were used. Both sensors were laid on the ground, one dorsally and the other ventrally to the carcass. Sensors were not protected in any way. Logging started at 6 a.m. of the first day, and 5-min logging intervals were used.

Degree-days (DD) were calculated with the formula DD = *T* − LT, where *T* is the mean daily temperature of the ground level and LT is a lower temperature threshold. For the purpose of this study, 0 °C was used as the lower temperature threshold, as this threshold is widely accepted in forensic decomposition studies [16, 22–29]. When *T* < LT, DD were set to zero. Accumulated degree-days (ADD) were calculated by adding together DD for a given interval.

### Data analyses

Treatment effects were tested with three-way ANOVA. Carcass mass, carcass clothing and blocks were main factors, with an interaction of carcass mass and carcass clothing included. ANOVA for repeated measure designs was used to test differences between classes of carcasses in TBS. TBSs were recorded at 50, 100, 150, 200, 250, 300 and 350 ADD.

Rate of active decay was defined after Matuszewski et al. [21] and was quantified from the daily mass loss for days with active decay. Changes in rate were analysed in *XY*-coordinate system with ADD on the *X*-axis (the temperature/time scale) and the distance-weighted least squares method for curve smoothing. Patterns of mass loss were analysed in the same way. In order to avoid values above 100 %, the locally weighted scatter plot smoothing method was used for curve smoothing.

The relationship between the average rate of active decay and carcass mass was analysed with non-linear regression. The logarithmic model [average rate of active decay = *b* + *a* × log_10_(carcass mass)] was fitted by using the Levenberg-Marquardt procedure.

In all analyses, 5 % level of significance was accepted. Calculations were made using Statistica 9.1 (StatSoft, Inc. 2010).

## Results

### Gross processes of decomposition

#### General pattern

All carcasses revealed the same gross processes of decomposition (bloating, active decay and advanced decay). Decomposition was mosaic irrespective of carcass mass and clothing.

#### Onsets

**Fig. 2:**
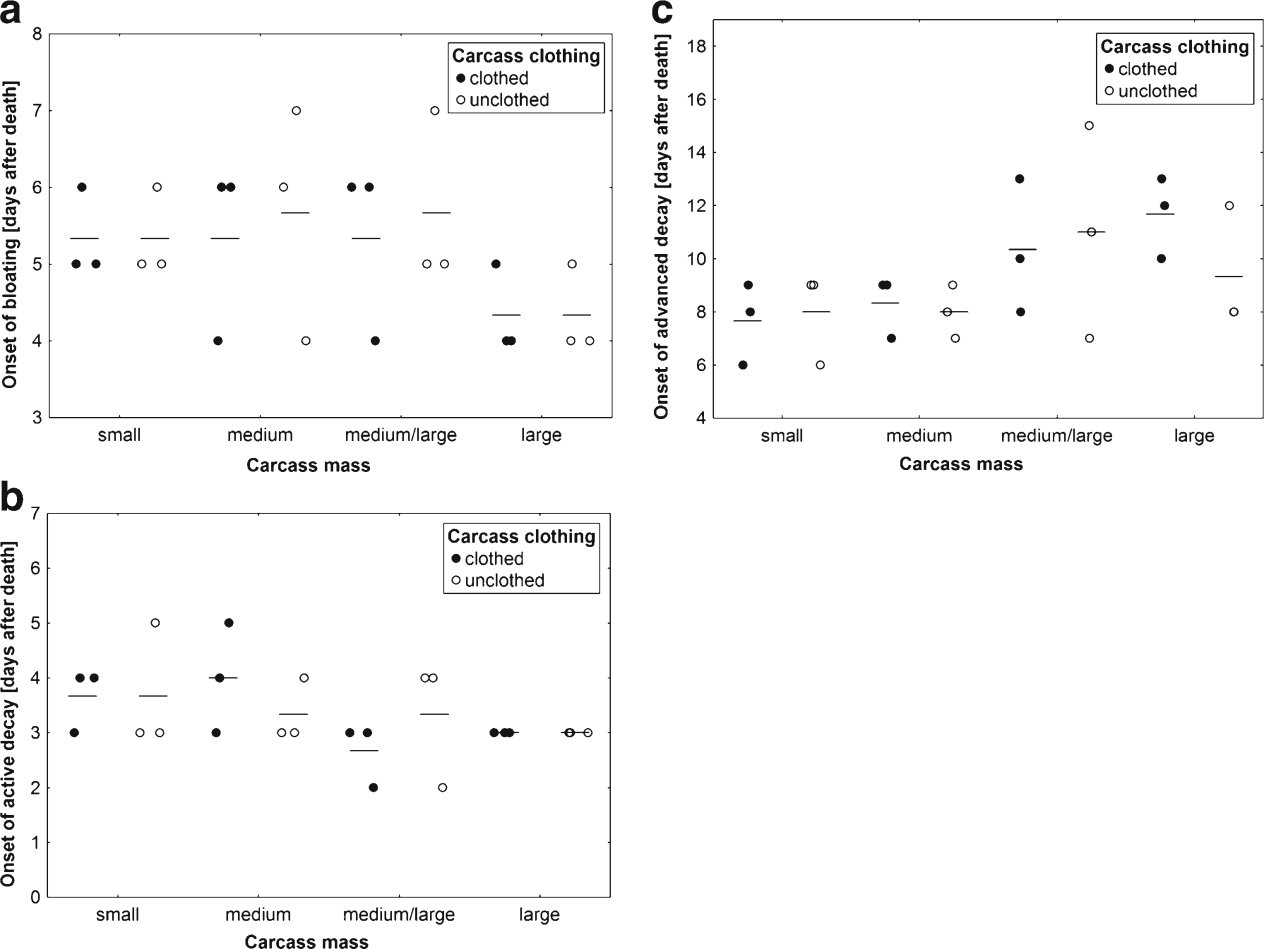
The effect of carcass mass and carcass clothing on the onset of bloating (**a**), the onset of active decay (**b**) and the onset of advanced decay (**c**). Carcass mass: small 5–15 kg, medium 15.1–30 kg, medium/large 35–50 kg and large 55–70 kg. *Horizontal lines* indicate the mean, while *open and closed circles* represent raw data

Carcass mass had a near-significant effect on the onset of bloating (ANOVA, *F*_3,14_ = 3.02, *P* = 0.06; Fig. 2a), no significant effect on the onset of active decay (ANOVA, *F*_3,14_ = 1.51, *P* = 0.26; Fig. 2b) and a significant effect on the onset of advanced decay (ANOVA, *F*_3,14_ = 4.17, *P* = 0.03; Fig. 2c). Bloating started earlier on larger carcasses (Fig. 2a), whereas advanced decay was earlier on smaller carcasses (Fig. 2c). Effects of carcass clothing and the interaction of effects were insignificant (ANOVA, *P* > 0.5; Fig. 2a–c).

#### Durations

**Fig. 3:**
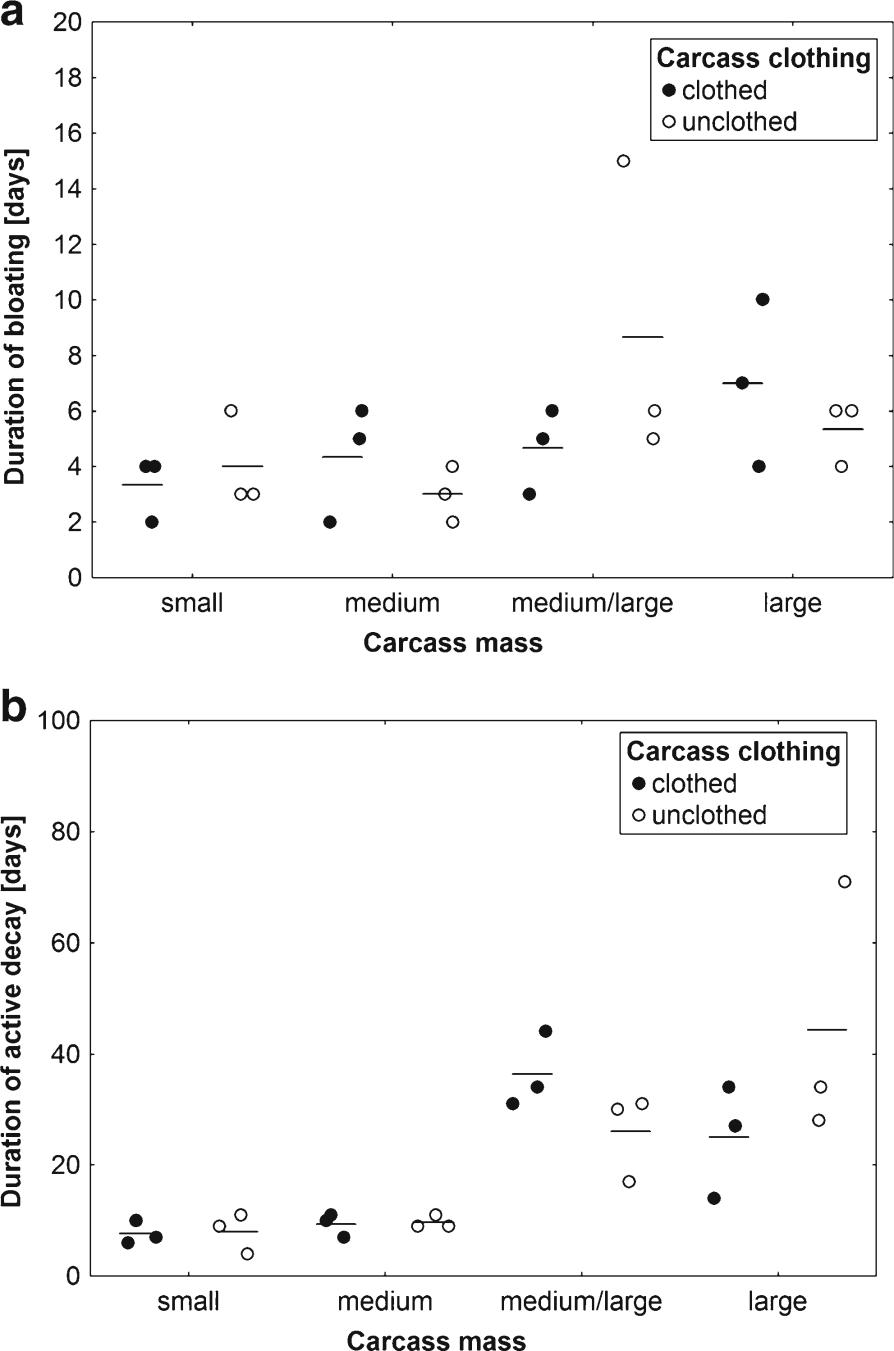
The effect of carcass mass and carcass clothing on the duration of bloating (**a**) and the duration of active decay (**b**). Carcass mass: small 5–15 kg, medium 15.1–30 kg, medium/large 35–50 kg and large 55–70 kg. *Horizontal lines* indicate the mean, while *open and closed circles* represent raw data

Carcass mass revealed a close-to-significant effect on the duration of bloating (ANOVA, *F*_3,14_ = 2.13, *P* = 0.14; Fig. 3a) and a significant effect on the duration of active decay (ANOVA, *F*_3,14_ = 12.83, *P* = 0.0002; Fig. 3b). Both processes lasted longer on larger carcasses (Fig. 3a, b). Effects of carcass clothing and the interaction of effects were insignificant (ANOVA, *P* > 0.1; Fig. 3a, b).

#### Pattern and rate of active decay

**Fig. 4:**
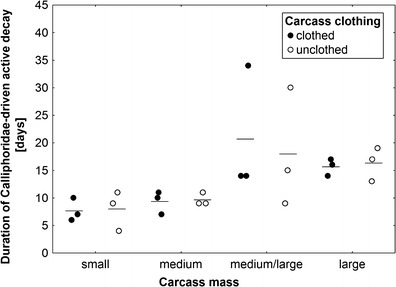
The effect of carcass mass and carcass clothing on the duration of active decay driven by larval blowflies (Diptera: Calliphoridae). Carcass mass: small 5–15 kg, medium 15.1–30 kg, medium/large 35–50 kg and large 55–70 kg. *Horizontal lines* indicate the mean, while *open and closed circles* represent raw data

**Fig. 5:**
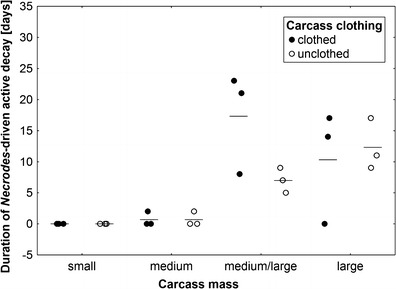
The effect of carcass mass and carcass clothing on the duration of active decay driven by larval *N. littoralis* (Coleoptera: Silphidae). Carcass mass: small 5–15 kg, medium 15.1–30 kg, medium/large 35–50 kg and large 55–70 kg. *Horizontal lines* indicate the mean, while *open and closed circles* represent raw data

Active decay was driven by larval blowflies (Diptera: Calliphoridae) on all carcasses, with larger carcasses revealing significantly longer duration of blowfly-driven active decay (ANOVA, carcass mass effect: *F*_3,14_ = 5.36, *P* = 0.01; carcass clothing effect: *F*_1,14_ = 0.02, *P* = 0.89, the interaction of effects: *F*_3,14_ = 0.11, *P* = 0.95; Fig. 4). Blowfly-driven active decay was regularly followed by active decay driven by larval *N. littoralis* L. (Coleoptera: Silphidae) in medium/large and large carcasses, and again, larger carcasses revealed a significantly longer duration of this type of active decay (ANOVA, carcass mass effect: *F*_3,14_ = 10.9, *P* = 0.0005; carcass clothing effect: *F*_1,14_ = 1.08, *P* = 0.32, the interaction of effects: *F*_3,14_ = 1.94, *P* = 0.17; Fig. 5).

**Fig. 6:**
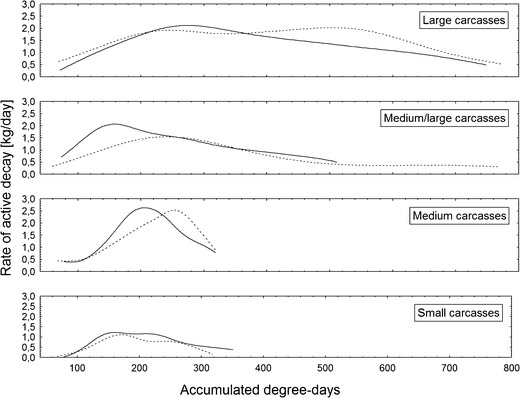
Changes in the rate of active decay during decomposition of clothed and unclothed pig carcasses of different mass. The distance-weighted least squares method for curve smoothing was used. Small carcasses 5–15 kg, medium carcasses 15.1–30 kg, medium/large carcasses 35–50 kg and large carcasses 55–70 kg. *Solid line* indicates unclothed carcasses, while *broken line* represents clothed carcasses

**Fig. 7:**
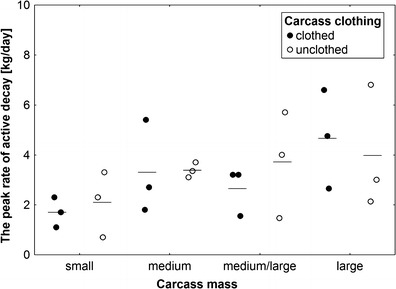
The effect of carcass mass and carcass clothing on the peak rate of active decay. Carcass mass: small 5–15 kg, medium 15.1–30 kg, medium/large 35–50 kg and large 55–70 kg. *Horizontal lines* indicate the mean, while *open and closed circles* represent raw data

**Fig. 8:**
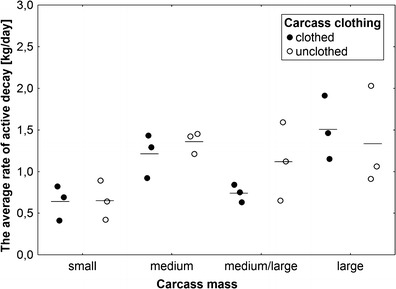
The effect of carcass mass and carcass clothing on the average rate of active decay. Carcass mass: small 5–15 kg, medium 15.1–30 kg, medium/large 35–50 kg and large 55–70 kg. *Horizontal lines* indicate the mean, while *open and closed circles* represent raw data

**Fig. 9:**
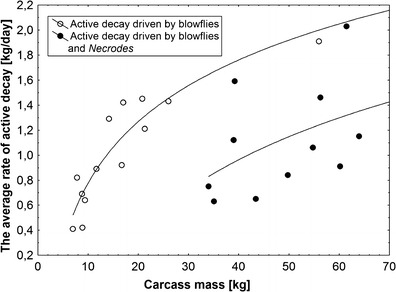
The relationship between carcass mass and the average rate of active decay for carcasses with active decay driven by larval blowflies (**a**) and carcasses with active decay driven by larval blowflies and larval *N. littoralis* (**b**). **a** The average rate of active decay = −0.86082 + 1.6365 × log_10_(carcass mass), *r* ^*2*^ = 0.85. **b** The average rate of active decay = −2.0844 + 1.90243 × log_10_(carcass mass), *r* ^*2*^ = 0.2

**Fig. 10:**
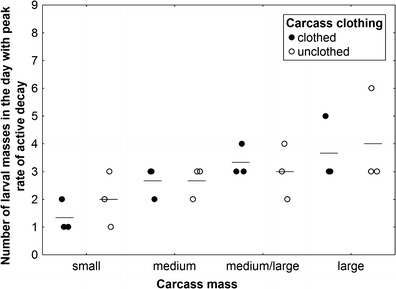
The effect of carcass mass and carcass clothing on the number of larval masses in the day with peak rate of active decay. Carcass mass: small 5–15 kg, medium 15.1–30 kg, medium/large 35–50 kg and large 55–70 kg. *Horizontal lines* indicate the mean, while *open and closed circles* represent raw data

Pattern of changes in the rate of active decay was different in different classes of carcass mass (Fig. 6). Most classes revealed one peak in rate (Fig. 6). The increase in rate was the fastest in medium carcasses and the slowest in small and large carcasses (Fig. 6). Although the peak rate of active decay was higher on larger carcasses (Figs. 6 and 7), these differences were not significant (ANOVA, *F*_3,14_ = 2.1, *P* = 0.15; Fig. 7). The decrease in rate was much slower on larger carcasses; the fastest decrease was recorded again in medium carcasses (Fig. 6). Carcass mass had a significant effect on the average rate of active decay (ANOVA, *F*_3,14_ = 5.9, *P* = 0.008; Fig. 8), with medium and large carcasses revealing higher rates than small and medium/large carcasses (Fig. 8). Effect of carcass clothing and the interaction of effects were insignificant (ANOVA, *P* > 0.5; Fig. 8). The average rate of active decay showed a significant, logarithmic increase with an increase in carcass mass, but only in the case of these carcasses on which active decay was driven solely by blowflies (nonlinear regression, *t* test for *a*, *t* = 7.8, *P* < 0.0001, Fig. 9). Carcasses on which blowfly-driven active decay was followed by *Necrodes*-driven active decay showed only a slight and insignificant increase in average rate with an increase in mass (nonlinear regression, *t* test for *a*, *t* = 1.5, *P* = 0.17, Fig. 9). Larger carcasses had more larval masses, and these differences were particularly demonstrable for the day with the peak rate of active decay (ANOVA, carcass mass effect: *F*_3,14_ = 6.82, *P* = 0.005; carcass clothing effect: *F*_1,14_ = 0.22, *P* = 0.64, the interaction of effects: *F*_3,14_ = 0.38, *P* = 0.77; Fig. 10).

#### Pattern of advanced decay

**Fig. 11:**
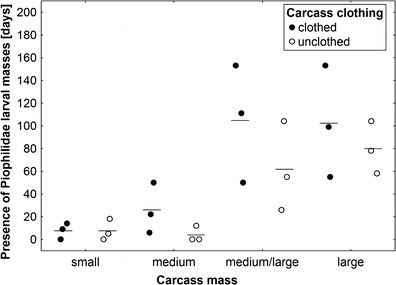
The effect of carcass mass and carcass clothing on the presence of Piophilidae larval masses. Carcass mass: small 5–15 kg, medium 15.1–30 kg, medium/large 35–50 kg and large 55–70 kg. *Horizontal lines* indicate the mean, while *open and closed circles* represent raw data

Much more biomass was left on larger carcasses after termination of active decay; consequently, advanced decay was more complex and lasted much longer on these carcasses, with regular and long-lasting presence of Piophilidae larval masses (ANOVA, *F*_3,14_ = 11.7, *P* = 0.0004; Fig. 11). There was also a close-to-significant effect of carcass clothing with clothed carcasses revealing longer presence of Piophilidae larval masses (*F*_1,14_ = 2.88, *P* = 0.11; the interaction of carcass mass and carcass clothing effects: *F*_3,14_ = 0.47, *P* = 0.71; Fig. 11).

### Pattern of mass loss

**Fig. 12:**
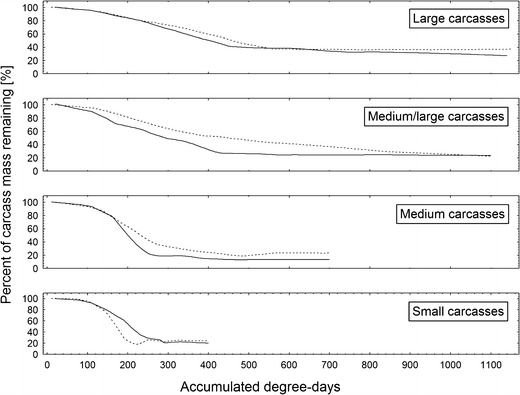
Carcass mass loss during decomposition of clothed and unclothed pig carcasses of different mass. The locally weighted method for scatter plot smoothing was used. Small carcasses 5–15 kg, medium carcasses 15.1–30 kg, medium/large carcasses 35–50 kg and large carcasses 55–70 kg. *Solid line* indicates unclothed carcasses, while *broken line* represents clothed carcasses

Classes of carcasses differed according to the pattern of mass loss (Fig. 12). Small and medium carcasses revealed a typical sigmoidal pattern, with an initial no-change phase, followed by a rapid decrease in mass and a final no-change phase with carcass mass stabilizing at about 20 % of its initial mass (Fig. 12). In the case of medium/large and large carcasses, there was a constant and slow decrease in mass, followed by the final no-change phase with no sigmoidal shape of the curve and carcass mass stabilization at about 20–40 % of its initial mass (Fig. 12). Moreover, the decrease in mass was slightly slower for clothed carcasses as compared to unclothed carcasses (with the exception of small carcasses for which the pattern was reversed) (Fig. 12).

### TBS

**Fig. 13:**
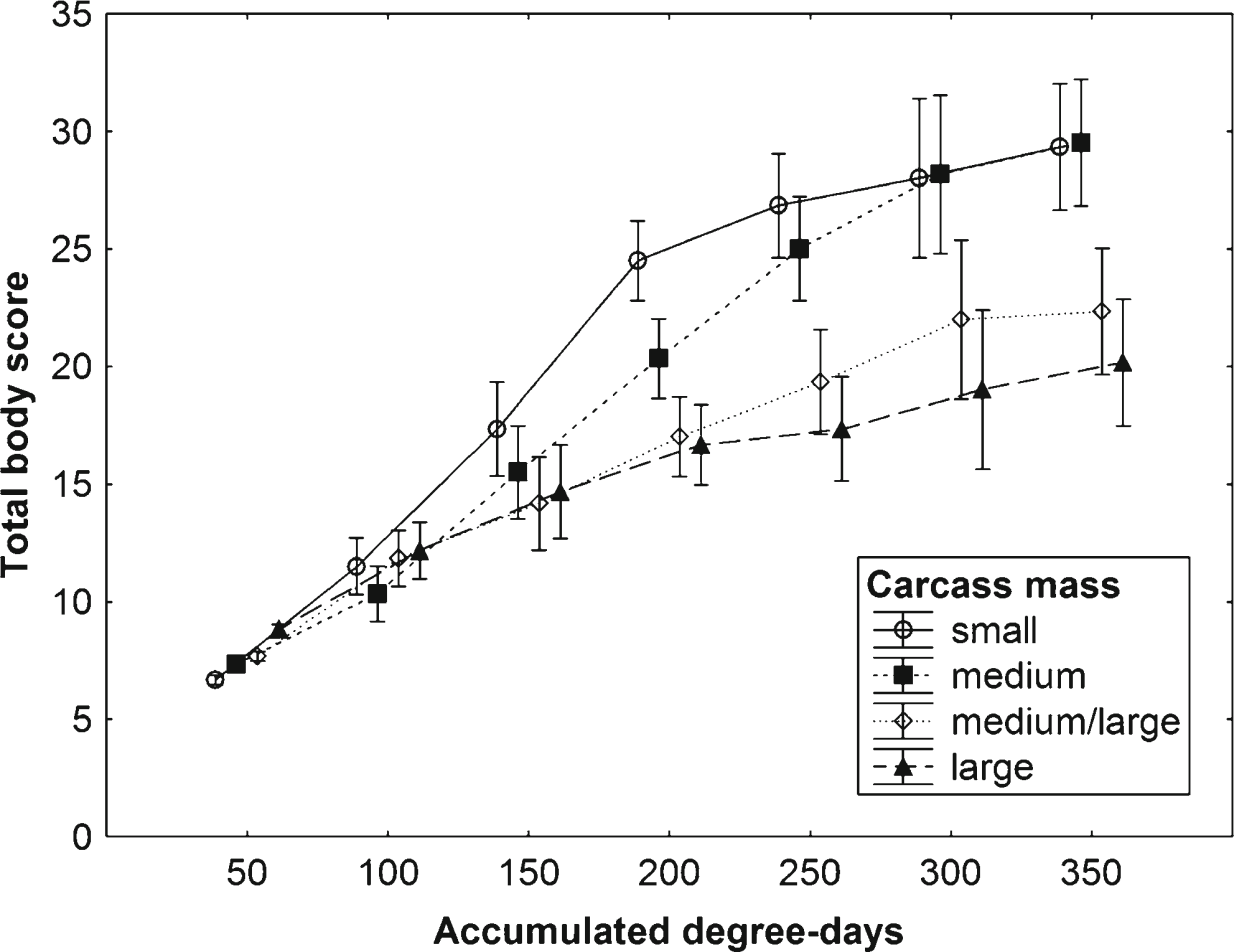
The effect of carcass mass and accumulated degree-days on total body score of pig carcasses. *Vertical bars* denote 0.95 confidence intervals. Carcass mass: small 5–15 kg, medium 15.1–30 kg, medium/large 35–50 kg and large 55–70 kg

Carcass mass and ADD had highly significant effects on TBS (repeated measures ANOVA, carcass mass effect: *F*_3,6_ = 21.9, *P* = 0.0012; ADD effect: *F*_6,36_ = 313.4, *P* < 0.0001, Fig. 13), effect of carcass clothing was insignificant (*F*_1,6_ = 0.09, *P* = 0.77) and the interaction of ADD and carcass mass effects was highly significant (*F*_18,36_ = 11.5, *P* < 0.0001, Fig. 13). Until about 100 ADD, larger carcasses gained higher scores than smaller carcasses; afterwards, the pattern was reversed and smaller carcasses were scored higher than larger carcasses (Fig. 13). These differences enlarged with the progress of decomposition (Fig. 13).

## Discussion

The primary result of the current experiment is a demonstration that carcass mass is a factor of key importance for decomposition, whereas the significance of carcass clothing is negligible. Carcass mass revealed significant effects in almost all analyses, whereas carcass clothing showed only a minor and inconsistent effect on the rate of mass loss and close-to-significant effect on the duration of Piophilidae larval masses. As for the importance of carcass clothing, current results are in line with the results of Kelly et al. [19]. A larger importance of clothing was suggested by Voss et al. [20], who revealed that dipteran larval masses were present for a longer period of time on clothed carcasses, and thereby, the wet decay stage was markedly prolonged on these carcasses. Current results only partially support this view, as there was no effect of clothing on the duration of active decay irrespective of insects forming larval masses (Figs. 4 and 5). On the other hand, clothed carcasses revealed longer presence of larval masses formed by Piophilidae (Fig. 11). These flies regularly colonize carcasses and human corpses late in decomposition [30], and their larvae feed on a substrate remaining after blowfly-driven or *Necrodes*-driven active decay. In Europe, *Stearibia nigriceps* (Meigen) is of particular forensic importance [9, 30–35]. It was found to oviposit on pig carcasses at the earliest 8 days following death, and in lower temperatures, this interval was much larger [36], which indicates that larval masses of this species are peculiar to carcasses in advanced decay. Current results thus support the view that the minor influence of carcass clothing is noticeable only in later portions of decomposition and, in particular, after the onset of advanced decay. It is however of importance that the current experiment involved only moderate clothing (Fig. 1), whereas heavy clothing may have more important effects on decomposition than the effects currently reported [37]. Heavy clothing can favour the putrefaction process and protect the corpse from scavenging, as was recently recorded in a mass disaster case in a marine environment [38]. Moreover, the minor importance of moderate clothing for decomposition, as demonstrated in this article, should not lead to a conclusion that carcass clothing is forensically unimportant. Even though it may have little effects on decomposition, clothing and accurate information about its kind and extent of are important for many other forensically relevant reasons [3, 37, 39].

Unexpectedly, carcass mass differently affected particular gross processes of decomposition. Bloating started significantly earlier and lasted slightly longer on larger carcasses, suggesting that putrefaction is more efficient in these carcasses probably due to larger and more complex microbial communities [40] and slower heat loss [41]. Active decay started in the same moment, regardless of carcass mass, but lasted much longer on larger carcasses. The peak and the average rate of active decay were distinctly lower in small carcasses; however, medium carcasses revealed a higher average rate than medium/large carcasses and only minimally lower than large pigs. Moreover, current results indicate that the average rate of active decay is clearly related to the pattern of active decay. Carcasses on which this process was dominated by blowflies revealed a logarithmic increase in rate with an increase in mass and relatively higher average rate as compared to carcasses on which active decay was driven by blowflies and *N. littoralis*. These results indicate that a multi-guild active decay is less efficient than active decay driven by one guild of insects. *N. littoralis* is a carrion beetle (Silphidae) which regularly colonizes large vertebrate carcasses in Europe [33, 34, 42–44]. It was also regularly sampled in human death cases [45]. Adult *N. littoralis* was found to appear on carcasses at the earliest of 2 days after death; however, in low temperatures, it usually appeared much later [46, 47]. In this study, it was very abundant on medium/large and large carcasses and the highest abundance of the adult *N. littoralis* usually coincided with the presence of large masses of feeding third instar blowfly larvae on which it voraciously fed. Accordingly, presence of large number of adult *N. littoralis* resulted in serious reduction of the number of feeding maggots and eventually lowered the average rate of active decay on carcasses with *Necrodes*-driven active decay. Similar effects were reported for carcasses infested with larval *Chrysomya albiceps* (Diptera: Calliphoridae), as this blowfly may seriously reduce larval masses formed by maggots of early-colonizing blowflies [42, 48]. It is also of importance that presence of blowfly larval masses and *Necrodes* larval masses may be separated by an interval with no clear larval masses, and this may further reduce the average rate of active decay. Therefore, current results indicate that active decay is less efficient on larger carcasses, and this is a consequence of a multi-guild and competition-related pattern of active decay in these carcasses. Moreover, it seems that such an effect is usually a case, when active decay is driven by insect guilds competing for carrion resources. As for the advanced decay, current results indicate that it starts significantly earlier on smaller carcasses, which is due to more efficient active decay on these carcasses. On the other hand, advanced decay was more complex and lasted longer on larger carcasses, which resulted from much more biomass being left on larger carcasses after termination of active decay. In summary, results of this study indicate that all gross processes of decomposition heavily depend on carcass mass; however, the effect of carcass mass on specific processes is different, and consequently, the combined effect on decomposition is more complex than previously assumed.

Previous results regularly demonstrated that mass loss in carcasses exposed on the ground follows a sigmoidal pattern [42, 49–52]. Most of these studies analysed mass loss in small- or medium-sized corpses; however, current results indicate that in larger carcasses (particularly above 50 kg), pattern of mass loss may be better represented by an exponential decay or by a join of two different linear functions.

When one looks globally at the decomposition, current results lead to another important conclusion—that rate of decomposition is strongly related to carcass mass, however not linearly. In a low mass range, it increases logarithmically with increasing mass, whereas at about 30 kg, it distinctly decreases and again at about 50 kg, it starts to increase. This pattern is strongly suggested by differences in average rate of active decay, differences in pattern of mass loss and partially by differences in the rate of TBS increase. As for the TBS, current results are generally consistent with this view. However, the difference in the rate of TBS increase between small and medium carcasses is unclear. The rate of increase between 100 and 200 ADD was higher in small than medium carcasses, whereas between 200 and 300 ADD, this pattern was reversed. The non-linear relationship between decomposition rate and carcass mass shows that seeming inconsistency between results of previous studies [12–17] was simply a consequence of analysing the effects in different ranges of carcass mass.

Surprisingly, differences in TBS between carcasses of different mass were dissimilar in initial and main portion of decomposition. Initially, higher scores were given to larger carcasses due to more efficient putrefaction in these carcasses, whereas from about 100 to 150 ADD, smaller carcasses were scored higher and this change resulted from more efficient active decay in small and medium carcasses. These results suggest that the pattern of changes in TBS is different in different classes of carcass mass. In larger carcasses, it is clearly logarithmic, whereas in smaller carcasses, it is slightly sigmoidal.

Moreover, current results indicate that decomposition-related methods for PMI estimation, for example TBS method of Megyesi et al. [22], may ignore cadaver clothing, but should not ignore cadaver mass. They also strongly suggest that carcass mass affects many entomological phenomena on carrion. Accordingly, a question arises in what way should taphonomic and entomological methods for PMI estimation account for these effects.
